# Adaptive Computing Optimization in Software-Defined Network-Based Industrial Internet of Things with Fog Computing

**DOI:** 10.3390/s18082509

**Published:** 2018-08-01

**Authors:** Juan Wang, Di Li

**Affiliations:** School of Mechanical and Automotive Engineering, South China University of Technology, Guangzhou 510641, China; itdili@scut.edu.cn

**Keywords:** fog computing, computing mode selection (CMS), IIoT, software-defined network (SDN)

## Abstract

In recent years, cloud computing and fog computing have appeared one after the other, as promising technologies for augmenting the computing capability of devices locally. By offloading computational tasks to fog servers or cloud servers, the time for task processing decreases greatly. Thus, to guarantee the Quality of Service (QoS) of smart manufacturing systems, fog servers are deployed at network edge to provide fog computing services. In this paper, we study the following problems in a mixed computing system: (1) which computing mode should be chosen for a task in local computing, fog computing or cloud computing? (2) In the fog computing mode, what is the execution sequence for the tasks cached in a task queue? Thus, to solve the problems above, we design a Software-Defined Network (SDN) framework in a smart factory based on an Industrial Internet of Things (IIoT) system. A method based on Computing Mode Selection (CMS) and execution sequences based on the task priority (ASTP) is proposed in this paper. First, a CMS module is designed in the SDN controller and then, after operating the CMS algorithm, each task obtains an optimal computing mode. Second, the task priorities can be calculated according to their real-time performance and calculated amount. According to the task priority, the SDN controller sends a flow table to the SDN switch to complete the task transmission. In other words, the higher the task priority is, the earlier the fog computing service is obtained. Finally, a series of experiments and simulations are performed to evaluate the performance of the proposed method. The results show that our method can achieve real-time performance and high reliability in IIoT.

## 1. Introduction

In the context of Industry 4.0, Internet of Things (IoT), cloud computing, Big Data and other advanced technology provides technical support for the development of intelligent manufacturing [1,2,3,4]. In a smart factory, humans, machines and things are connected by Industrial IoT (IIoT) [5,6,7,8]. The information on the environment, equipment and personal is collected by intelligent terminals such as sensors, handheld devices and wearable devices. The cloud provides a service platform for data processing and data analysis [9]. Cloud computing provides a solid foundation for the realization of intelligent manufacturing. With the explosive growth of terminal devices, the scale of IIoT is increasingly amplified. Massive data transmission will cause network congestion and network bandwidth bottlenecks have become an obstacle that baffles the development of cloud computing; even worse, network delay reduce the Quality of Service (QoS) for cloud computing [10,11,12].

In the process of intelligent manufacturing, to satisfy the small batch production of individualized products, an increasing number of computation-intensive and delay-sensitive tasks must be executed in an intelligent factory, which brings new problems and challenges to the cloud computing [13]. It is difficult to have real-time knowledge of the operating equipment status for the cloud computing. In the smart factory, production scheduling is a real-time multitask and multiobject application and the cloud has difficulty in guaranteeing the QoS, such as real-time performance and reliability [14]. The augmented reality applications typically require a response time of approximately 10 ms, which is difficult to achieve using the cloud solution with a typical end-to-end latency of hundreds of milliseconds [15].

To make up for the inadequacy of cloud computing, a new computing mode-fog computing is proposed in the field of manufacturing [16]. Fog nodes with plenty of computing resources and storage resources are located near the network edge and terminal equipment or user can offload cloud applications to the fog nodes [17]. Fog computing has a low latency, high reliability, energy savings and safety, which is difficult to realize with a remote cloud [18,19,20]. There are three types of computing modes that exist in IoT systems: local computing, fog computing and cloud computing. The performances and characteristics are described in [21]. Here, the system with three computing modes can be called a fog computing system. Computing Mode Selection (CMS) is the key technology for the fog computing system and it should have the ability for adaptive computing for all types of tasks.

Many studies have investigated the issues that concern cloud computing, fog computing or the combination of the two types of modes. Usually, the computational capacity of terminal devices is ignored and tasks are offloaded to fog nodes or the cloud indirectly for most of the studies in the literature. In an intelligent plant, terminal devices include robotic arm, conveyor belt, Automatic Guided Vehicle (AGV), Computer Numerical Control (CNC), sensors and hand-held terminals and so forth. These devices have a certain computing power and storage capacity and they can complete some simple applications by themselves. However, some terminal devices such as ordinary sensors do not have computational ability, so they must be connected with agents (e.g., Raspberry Pi) for sensor data preprocessing. The research fields of fog computing are mainly distributed in mobile cellular networks, vehicular networks, healthcare and other applications and have made great progress. However, the research on fog computing in intelligent manufacturing is still in its infancy. In addition, the QoS of IIoT is different from IoT: the IIoT emphasizes real-time processing and reliability compared to the IoT, which emphasizes the throughput and packet loss rate [22].

The cloud computing is a centralized processing architecture. All of the tasks are completed on the cloud and all of the raw data obtained must be transmitted to the cloud. Thus, this approach will consume a large amount of network bandwidth. With the increasing demand of applications, the network traffic will increase enormously, which will result in the interruption of services, network delay and other issues. Therefore, to improve the real-time performance of the cloud computing, edge servers are deployed in a smart factory and a fog computing system is built for task processing. Software-Defined Network (SDN) is regarded as a crucial technique for IIoT and thus, we establish a Software-Defined IIoT framework. In our work, a CMS module and a task execution sequence adjustment module are designed in the SDN controller and an optimal computing mode decision for each task is generated by running a CMS algorithm. The execution sequence of delay-sensitive tasks depends on their priority; in other words, the higher the task priority is, the earlier the task processing. The contributions of this paper are as follows:

1. A fog computing system is established in the Software-Defined IIoT. The computing resources of local devices, fog nodes and the cloud can be utilized effectively for task processing. An adaptive selection and task priority (ASTP) method is proposed in this paper.
2. A CMS module is designed in the SDN controller and a mode selection algorithm is proposed. The mode selection algorithm can choose an optimal computing mode for each task with real-time performance.
3. To promise and improve the QoS of a smart factory, a task execution sequence based on task priority is introduced. The SDN controller makes a flow table according to the task priorities and then, these flow tables are sent to the assigned switches. The execution sequence of the tasks that require fog services is determined.
4. We perform simulation results to evaluate the proposed method. The results demonstrate that the proposed method outperforms the conventional methods in terms of the average delay, total delay, reliability and satisfaction.

The remainder of this paper is organized as follows. The related work is presented in Section 2. The IIoT framework with SDN and fog computing system is introduced in Section 3. In Section 4, a method of CMS and execution sequence for the tasks with the fog computing system is studied. The simulation results are discussed in Section 5. In Section 6, a brief conclusion is given.

## 2. Related Work

Fog computing and cloud computing are interdependent on each other and they form a service continuum between the cloud and the terminal devices of the IIoT. Fu et al. [23] designed a flexible and economic framework by integrating fog computing and cloud computing and the problem of storage and searching for secure data can be solved quickly and effectively. In the field of healthcare, Muhammad et al. [24] proposed a smart healthcare framework that uses fog computing and cloud computing. A voice disorder assessment and treatment using a deep learning approach was developed. The voice sample goes to the fog computing for the initial processing and then, the data that are preprocessed are sent to a core cloud for further processing. Experimental results verified that the accuracy and sensitivity improved greatly.

Mobile Edge Computing (MEC) is a typically paradigm of edge computing and this technology is applied in smart cities and smart transport fields with low-latency and high-reliability. Taleb et al. [25] utilized MEC to enhance the user’s experience of video streaming in smart cities. Through a smart MEC architecture, an important solution for reducing the core network traffic and ensuring ultralow latency was proposed. Liu et al. [26] proposed an SDN-enabled network architecture that was assisted by MEC. The results have shown that the architecture can meet application-specific requirements and maintain good scalability and responsiveness. In [27], the authors proposed a MEC-based system for charging station working efficiently with a Big Data-driven planning strategy. Computing offloading is a crucial technique for fog/edge computing and cloud computing. In [28], the authors studied the hybrid computation offloading problem while considering the diverse computing and communication capabilities of two types of offloading destinations, that is, cloud and fog. In [29], the tradeoff between the latency and reliability in the task offloading to mobile edge/fog computing is studied and the user equipment is partitioned by dividing a task into subtasks and offloading them to multiple nearby fog nodes in sequence. Three algorithms were designed to solve the optimization problem to jointly minimize the latency and offloading failure probability. Numerical simulation results show that the proposed algorithms strike a good balance between the latency and reliability in ultra-Reliable Low Latency Communications (uRLLC). Shih et al. [30] introduced the Fog-Radio Access Network (F-RAN) architecture, which brings the efficient computing capability of the cloud to the fog of the network. By distributing computing-intensive tasks to multiple F-RAN nodes, F-RAN has the potential to meet the requirements of those ultralow latency applications. Hu et al. [31] proposed a fog computing-based face identification and resolution method. To improve the processing efficiency and reduce the network transmission, some computing overhead was offloaded from a cloud to network fog devices.

SDN is an emerging network paradigm that brings new insights and has high potential to improve the agility, reliability, scalability and latency performance [26]. In [32], the authors proposed SDN for fog computing. A clear collaboration model is proposed for the SDN-fog computing interaction through practical architectures and the SDN-related mechanisms can feasibly operate within the fog computing infrastructures. Bi et al. [33] proposed a novel SDN based fog computing architecture by decoupling the mobility control and data forwarding. Under the proposed architecture, efficient signaling operations were designed to provide seamless and transparent mobility support to mobile users and the authors presented an efficient route optimization algorithm by considering the performance gain in the data communications and system overhead in mobile fog computing. Li et al. [22] proposed an adaptive transmission architecture with SDN and fog computing for IIoT. Data streams were divided into two groups and two different strategies were made for low-deadline and high-deadline situations. The results demonstrate that the proposed method outperforms the conventional method.

Recently, fog computing has acquired more and more attention in the industrial field and the most typical representation is in manufacturing. Georgakopoulos et al. [11] proposed a roadmap based on IoT and edge cloud computing for manufacturing. Wan et al. [14] utilized fog network nodes for energy-aware load balancing and scheduling in a smart factory. An energy-aware load balancing and scheduling method is proposed based on fog computing. Experimental results showed that the proposed method provides optimal scheduling and load balancing for mixed work robots. Ashjaei et al. [16] proposed a platform that uses fog computing to enhance smart maintenance management in a smart factory. Debrito et al. [34] discussed fog computing and its application paradigm in a smart factory and they concluded that programmable fog nodes make point-to-point communication between devices autonomous. In [35], the authors developed a prototype to explore the use of IoT devices that communicate with a cloud/fog-based controller. Mitigation mechanisms were applied to address the delays and jitter that are caused by the networks when the controller is offloaded to the fog or cloud. In this paper, the ASTP method is proposed and implemented in the IIoT fog computing system platform. Most literature did not consider dynamic resource while selecting mode. Through the centralized control feature of SDN, the proposed framework is capable of computing mode adaptive selection. The task priority is used for task execution sequence adjustment, so that the real-time performance of fog computing can be improved. The proposed ASTP method is suitable for processing industrial tasks in scalable and flexible fog environment.

## 3. System Model and Problem Formulation

In this section, a fog computing system architecture based on Software-Defined IIoT is set up, we describe the system model. The delay model of task processing under three different computing modes are formulated.

### 3.1. System Architecture and System Model

**Figure 1:**
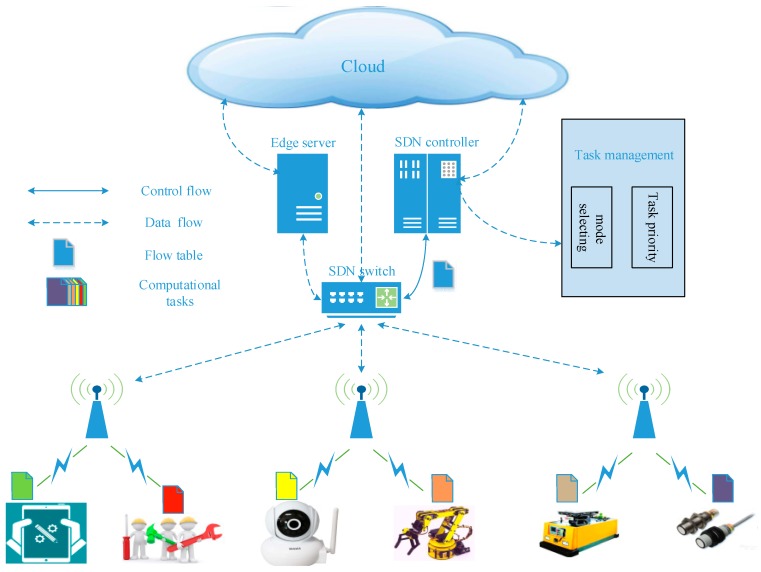
System architecture of Software-Defined IIoT based on fog computing in a smart factory.

In a smart factory, the manufacturing level has progressed greatly. Computational tasks in the productive process become increasingly complex, which brings about problems and challenges for terminal devices because of insufficient computing resources. However, cloud computing has been developed and proven to be effective for computation-intensive task processing. The cloud system architecture is limited by the constraints of the network bandwidth, communication delay, reliability and security and therefore, the cloud computing cannot promise the QoS of a productive system. To increase the flexibility, scalability and the real-time nature of the system, SDN technology and fog computing technology are integrated into the cloud computing system and the proposed system architecture as shown in Figure 1. The cloud, fog nodes and other terminal devices are connected by the communication infrastructure.

The system architecture can be divided into three layers: terminal devices layer, fog computing layer and cloud computing layer. The terminal devices layer is mainly responsible for industrial production, data acquisition and data transmission. The fog computing layer is mainly responsible for processing real-time tasks in the edge servers, through deploying the SDN controller to optimizing the CMS and execution sequence. The cloud layer is mainly responsible for processing non-real-time and computation-intensive tasks. The overall architecture of the fog computing system, for computational tasks, has three computing modes to choose from; in other words, the computational tasks can be executed by terminal devices, edge server or cloud. In this system, there is a set of terminal devices in a smart factory, which is denoted Ñ = {1, 2, …, *n*, …, N} and each device has a computation task to be completed with a certain delay constraint. The task attribute of device *n* is described by *J_n_* = {*D_n_*, *C_n_*, *T_n,max_*}, *n*∈Ñ. For task *J_n_*, where *D_n_* is the size of the input data (in bits), *C_n_* denotes the calculation amount (in CPU cycles) that is required to accomplish the task, which depends on the computational complexity of the task. *T_n,max_* is the maximum tolerable latency (in second) requirement of task *J_n_*. In this paper, we take a smart factory as the research object and fog computing service is supplied by an edge server, while cloud computing service is supplied by the cloud. Assuming that each task cannot be divided and the edge server can process simultaneously many computational tasks. The delay-sensitive task can be transmitted to the edge server through the Local Area Network (LAN); the compute-intensive task can be transmitted to the cloud through the Wide Area Network (WAN). In an industrial scene, especially in the intelligent manufacturing, real-time performance is the key performance indicator of the intelligent manufacturing system and perhaps even at the expense of energy consumption to improve the real-time performance and thus, this article does not consider energy consumption problems.

### 3.2. Delay Model of Tasks under Different Computing Modes

#### 3.2.1. Local Computing

Let *f_n_* be the computation ability of device *n* (in CPU cycles/s) and thus, the execution time of task *J_n_* is

*T_n_*_,*L*_ = *C_n_*/*f_n_*, (1)

#### 3.2.2. Fog Computing

If task *J_n_* chooses the fog computing mode, then terminal device *n* must transmit the input data *D_n_* to the fog server through the shared wireless links. After all of the input data *D_n_* is received, task *J_n_* will be processed in the edge server. The data transmission rate [36] of terminal device *n* to the edge server is

*r_n,E_* = *θ_n_·B*·log_2_(1 + *P_n,E_*·*g_n,E_*/*θ_n_*·*N*_0_·*B*), (2)

where *θ_n_* denotes the normalized assigned portion of the bandwidth to terminal device *n*. *B* is the total radio bandwidth and *P_n,E_* is the transmission power of terminal device *n* when transmitting data to the edge server, which is determined by the fog server through power control mechanisms and the maximum value is *P_n,max_*. Here, *g_n,E_* is the channel gain between terminal device *n* and the fog server, while *N_0_* is the channel noise power.

Thus, in the fog computing mode, the execution time of task *J_n_* consists of two sections: one is the transmission time and the other is the computing time. *f_n_,_E_* denotes the computational resource allocated by the edge server.

*T_n,E_* = *D_n_*/*r_n_*_,*E*_ + *C_n_*/*f_n_*,*_E_*, (3)

#### 3.2.3. Cloud Computing

Typically, those tasks that have no real-time demand and a large amount of calculation choose the cloud computing mode and are sent to the cloud and rely on a combination of the access network and core network. The execution time of the tasks is composed of three parts, the access network transmission time, the core network transmission and the cloud processing time.

*T_n_*_,*C*_ = *D_n_*/*r_n_*_,*E*_ + *D_n_*/*B_E_*_,*C*_ + *C_n_*/*f_C_*, (4)

where *B_E,C_* is the network bandwidth between the edge server and the cloud and *f_C_* is the computing ability of the cloud. It is worthwhile to note that the output data size of calculation results is smaller than the input data and in addition, the backhaul network resource is sufficient and thus, the return time of the calculation results is considered negligible.

### 3.3. Problem Formulation

According to (1), (3) and (4), the execution time of task *J_n_* is expressed as

*T_n_* = *a_n_*·*T_n_*_,*L*_ + *b_n_*·*T_n_*_,*E*_ + *c_n_*·*T_n,C_*, (5)

where *a_n_*, *b_n_*, *c_n_*, are the mode selection decisions of task *J_n_*, *a_n_*∈{0, 1}; *b_n_*∈{0, 1}; *c_n_*∈{0, 1}. *a_n_* + *b_n_* + *c_n_* = 1. Thus, the total delay of all of the tasks is formulated as follows:

*T_total_* =∑(*a_n_*·*T_n_*_,*L*_ + *b_n_*·*T_n_*_,*E*_ + *c_n_*·*T_n_*_,*C*_), (6)

(7) $$\begin{array}{cc} (P1): & \min\;T_{\mathrm{total}}\\ s.t.\;(C1): & a_{n},b_{n},c_{n}\in \{0,\;1\},\;\forall n\in Ñ,\\ (C2): & a_{n}+b_{n}+c_{n}=1,\;\forall n\in Ñ,\\ (C3): & 0<\theta_{n}\leq 1,\;\forall n\in Ñ,\\ (C4): & \sum \theta_{n}\leq 1,\;\forall n\in Ñ,\\ (C5): & 0\leq P_{n,E}\leq P_{n,max},\;\forall n\in Ñ,\\ (C6): & T_{n}\leq T_{n,max},\;\forall n\in Ñ,\\ (C7): & 0\leq f_{n,E}\leq f_{E},\;\forall n\in Ñ,\\ (C8): & \sum f_{n,E}\leq f_{E},\;\forall n\in Ñ, \end{array}$$

where (C1) and (C2) are the constraints on the mode selection decision of each task, namely, each task can choose only one computing mode; (C3) and (C4) are the constraints on the bandwidth allocation; (C5) is the transmit power constraint of device *n*; and (C6) indicates that the execution time of each task should not exceed its tolerable deadline; (C7) and (C8) are the constraints on the edge server resource allocation.

## 4. Adaptive Computing Optimization Method

In an IIoT environment, both the terminal devices and tasks are heterogeneous. Terminal devices through a computing mode selector to decide which computing mode should be selected for each task. Terminal devices choose the optimal computing mode to minimize the computing cost. In this section, we introduce the CMS mechanism as well as task execution sequence adjustment mechanism for the terminal devices. Then, we design the computing mode selector using a computing cost minimization indicator and obtain the optimal computing mode strategy of the mobile terminals.

### 4.1. Computing Mode Selection Mechanism

**Figure 2:**
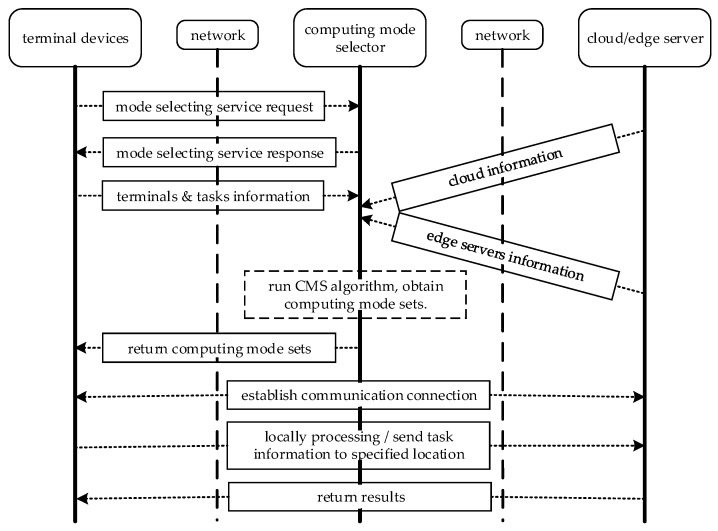
Signaling process for CMS.

In this section, we design a CMS module in the SDN controller. There are three computing modes that can be selected for each terminal device; they are local computing, fog computing and cloud computing. Different computing modes have different advantages, performance and features of the three computing modes. In the IIoT systems, tasks can be classified into offload tasks and unloadable tasks. The unloadable tasks represent the tasks that must be processed locally by the terminal devices. The offload tasks represent the tasks that can be processed by edge servers and cloud severs. Taking the smartphone as an example, for some applications such as calculators and notepads, the computation amount of these applications is relatively small and the results can be obtained easily by the smartphone. The usage of applications has nothing to do with the network state of the smartphone; these applications are called simple applications or unloadable applications. Legacy equipment deployed in the intelligent production line have poor function, in order to improve the computing power and storage capacity of the legacy equipment, usually an intelligent agent can be connected with the traditional equipment. For example, there some traditional equipment in a smart factory and their functions are simple and lack intelligence, while a Raspberry Pi can address complex applications such as sensory data preprocessing. However, in certain applications, such as production scheduling, operations and maintenance of production lines and remote monitoring of production processes, these applications need a synergy of intelligent devices and network devices. Thus, we call these applications smart applications. Smart applications must be accomplished with the aid of an edge server or cloud server. The main function of the CMS module is to select the best computing mode for different applications. The workflow diagram of CMS is illustrated in Figure 2.

The main working process of CMS is as follows. First, the terminal devices send a mode selection service request to the SDN controller when a task must be processed by terminal devices. After the terminal devices receive a service response, the terminal devices transmit the corresponding information to the SDN controller, which includes the computation ability of the devices and the basic information of the tasks, such as the data amount, calculation amount and maximum tolerance time. Then, the edge server and cloud send their information to the SDN controller, with information that includes the computation capacity, transmission power and network bandwidth. Second, to realize the real-time processing of the tasks, the CMS algorithm is run within the SDN controller. An optimal computing mode is obtained for each task. The computing mode sets are listed and *Φ_L_* denotes the local computing mode set; *Φ_E_* denotes the fog computing mode set; and *Φ_C_* denotes the cloud computing mode set. Three computing mode sets are sent to smart terminal devices. Third, for *Φ_L_* the tasks are processed directly within the terminal devices; for *Φ_E_* and *Φ_C_* smart terminal devices must establish communication connections with corresponding computing resources. The detailed information of the tasks is offloaded to the edge server or cloud. Lastly, after completion of the task computing, the edge server or cloud send the results to the terminal devices.

### 4.2. Task Execution Sequence Adjustment

The tasks selecting the fog computing service usually have a low delay requirement. An advanced execution sequence adjustment mechanism can improve the real-time performance. In this paper, the task priority is used for tasks execution sequence adjustment. With the concept of task priority, the overall QoS of the fog computing system can be improved. For example, a real-time IIoT application such as production process monitoring is assigned a higher priority, while other applications that consume more resources, such as multimedia peer-to-peer downloading, can be assigned a lower priority in such a way that the whole real-time performance of the manufacturing system can be improved.

In this paper, the task priority is analyzed from two attributes. The first attribute is the real-time level of the tasks and the second attribute is the computational requirements of the tasks. If the real-time level of a task is high and the computational requirement of the task is small, we define the task priority as high and in contrast, if the real-time level of a task is low and the computation amount of the task is large, the task priority is low. The maximum tolerance time represents the real-time level of the task; the smaller the maximum tolerance time is, the higher the real-time level. The calculation amount represents the complexity of the task; the smaller the calculation amount is, the lower the complexity. Here, we define the real-time intensity and complex intensity as task priority factors.

**Definition** **1:** *The set Ω of task priority factors is a set of pairs (α, β), where α denotes the real-time intensity of the tasks and β denotes the complex intensity of the tasks.*

*Then, the real-time intensity value of task i is formulated as*:

α*_i_* = *T_i,max_*/∑*T_i,max_*, (8)

*where T_i,max_ is the maximum tolerance time of task i, i* ∈*Φ_E_ and Φ_E_ denotes the set of tasks that choose the fog computing service.*

*Then, the complex intensity value of task i is formulated as:*

β*_i_* = *C_i_*/∑*C_i_,* (9)

*where C_i_ is the calculation amount of task i, i* ∈*Φ_E_ and Φ_E_ denotes the set of tasks that choose the fog computing service.*

*Hence, according to (7) and (8), the task priority is defined by:*

*p_i_* = *μ*_1_·α*_i_* + *μ*_2_·β*_i_*, (10)

*where μ*_1_, *μ*_2_ ∈[0, 1], *μ*_1_ + *μ*_2_ = 1 *denote the weights of the real-time intensity and complex intensity for task i, respectively. The smaller the value is for p_i_, the higher the task priority and task i obtains fog computing services earlier than other tasks.*

**Figure 3:**
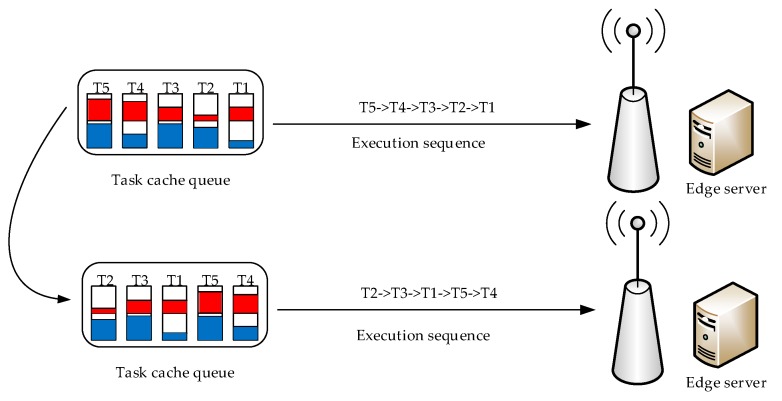
The execution sequence before and after using the task priority.

The top part of Figure 3 represents the task execution sequence based on a conventional mechanism, usually the first-come-first-processing mechanism. The bottom part is a novel execution sequence proposed in this paper. The red area denotes the real-time level of the tasks; the larger the red area, the higher real-time level of the task. The blue area denotes the computation amount of the tasks; the larger the blue area, the more of task computation amount. The real-time performance is the key performance indicator for industrial applications, when in the face of multitask processing, the real-time requirement of the tasks is considered first and thus, the real-time weight of the tasks is usually set high. In particular, for two or more tasks that have the same real-time level, the task with a low-complexity intensity is executed first. The reason is that the task with low complex intensity occupies fewer computation resources and thus, there will be remaining more computational resources for new incoming tasks with higher priority.

## 5. Simulation Results and Discussion

In this section, simulations are conducted to evaluate the performance of the proposed method. First, we describe the simulation setup, the performance metrics, the reference methods and the emulation scenarios. Then, the evaluation results are presented and discussed from various perspectives.

### 5.1. Simulation Setup

We developed the simulation framework and realized the proposed algorithm in the MATLAB environment. We set up a fog computing system in the IIoT based on the SDN. The main parameters values of the simulation Table 1. This paper takes five typical applications for performance evaluation and the details of the applications are shown in Table 2, the maximum tolerance time and calculation amount of each application are listed, the task priority of application is calculated. The hard real-time requirements are that if the task cannot be completed within the maximum tolerance, then the task failed.

### 5.2. Performance Metrics and Reference Methods

To evaluate the performance of the proposed method ASTP, we introduce the following performance metrics:

1. *Total Time Delay:* The total time delay represents the time needed for all of the applications services to be completed from the task requests to the return of the results.
2. *Average Time Delay:* The average time delay represents the time needed for each application service to be completed from the task request to the return of the results.
3. *Reliability:* The reliability represents the execution effect of the applications. The number of task failed is smaller and the reliability of the system is higher.
4. *Satisfaction:* The satisfaction represents the evaluation of the system according to the QoS. For a real-time application, if it is completed as soon as possible in the maximum tolerance time, the satisfaction is high.

We compared the proposed method ASTP with the following methods:

- Computing mode random selection and execution sequence based on conventional mechanism RSCM, wherein the computing mode of each task is randomly assigned. The tasks that selected the fog computing mode were completed according to the first-come-first-processing mechanism.
- Computing mode random selection and execution sequence based on task priority RSTP, wherein the computing mode of each task is randomly assigned. For these tasks that selected the fog computing mode, first the task priority should be calculated and then, the tasks were completed according to the task priority.
- Computing mode adaptive selection and execution sequence based on the conventional mechanism ASCM, wherein the mode selection module chooses the best computing mode for each task. The tasks that selected the fog computing mode were completed according to the first-come-first-processing mechanism.

### 5.3. Evaluation Results

**Figure 4:**
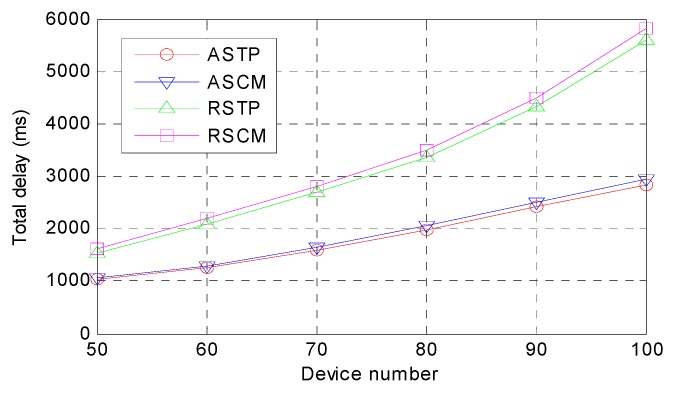
Comparison of the total time delay.

*Total Time Delay.* The total time delay of different methods for their best performance in terms of the time delay is presented in Figure 4, which demonstrates that the total time delay increases with an increase in the device amount for all of the methods. However, it is obvious that ASTP outperforms other methods in this metric with different device amounts. The reason is that the ASTP method can adaptively select an optimal computing mode for every device from the perspective of real-time performance. Both RSTP and RSCM randomly select computing mode for each device; those devices that have low-latency and compute-intensive tasks may be assigned to cloud computing, local computing or fog computing and thus, the total time delay is more than the other two methods. For ASTP and ASCM, although the computing mode of these two methods are the same, the task execution sequence of them are different. ASTP makes a novel execution sequence for the tasks according to the task priority, while ASCM still adopts the conventional execution sequence. Overall, their total time delay difference is very small. Similarly, for RSTP and RSCM, the computing modes of these two methods are randomly selected for the devices and thus, the real-time responses of the applications cannot be guaranteed.

**Figure 5:**
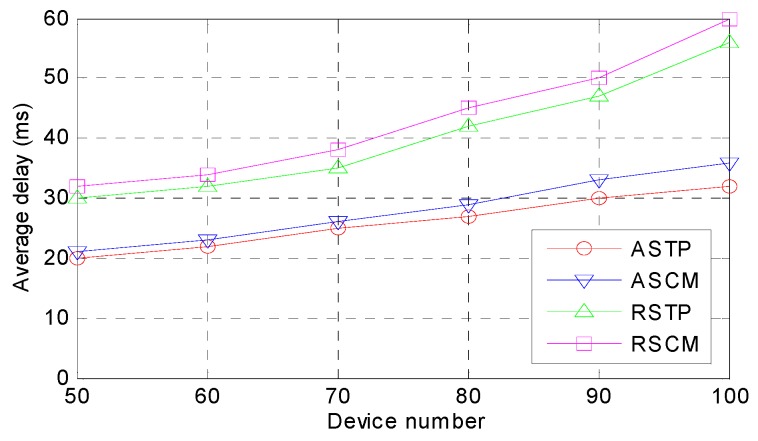
Comparison of the average time delay.

*Average Time Delay.* The average time delay in the four methods for the different device amounts is shown in Figure 5. In general, the average time delay of every method will increase slightly with an increasing number of devices. This trend is due to the optimization computing mode of ASTP and ASCM through the mode selection module and the average time delay of ASTP and ASCM is much less than the average time delay of RSTP and RSCM. For ASTP and ASM, although the CMS methods of the devices are the same, the execution sequences under the fog computing mode are different and thus, the ASTP achieves better performance than the ASCM. Similarly, the RSTP and RSCM are the same.

**Figure 6:**
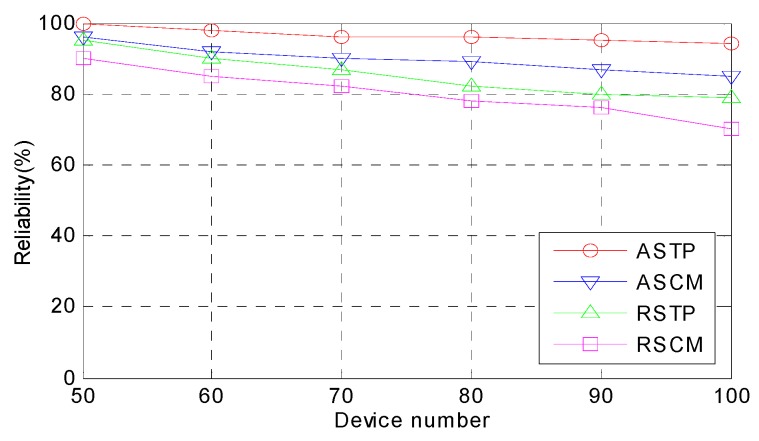
Comparison of the reliability.

*Reliability.* This performance metric is a great index for evaluating how successful is the task processing from the computing resources for the task. The failure rate of the tasks that are executed is lower and the reliability of the tasks is higher. As seen from Figure 6, with an increase in the number of devices, the reliability of the four methods decreases. In the ASTP method, with its unique mode selection algorithm and novel execution sequence algorithm, the reliability of the ASTP is higher than that of the other three methods. Compared to ASTP, RSCM is unsatisfactory. Random assigning of the computing mode and using the traditional execution sequence makes the computation-intensive and delay-sensitive tasks fail. The QoS of the system is then greatly reduced. When the device volume reached 100, the reliability of RSCM dropped to approximately 70%. Therefore, an efficient CMS and advanced execution sequence greatly improves the reliability of the system.

**Figure 7:**
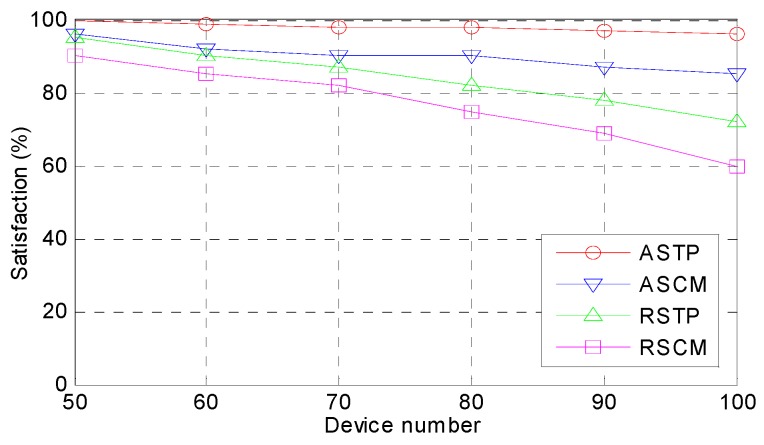
Comparison of satisfaction.

*Satisfaction.* The satisfaction from the different numbers of devices with the different methods is presented in Figure 7. Satisfaction is a comprehensive index for a system; from the perspective of real-time analysis, the application service is provided in a timelier fashion and the user satisfaction is higher. In a system with limited resources, however, the more service requests are, the larger the amount of pressure on the service providers and the user satisfaction would drop. How to provide more and better applications services by using the limited resources is an enormous challenge for current researchers. In this paper, when the number of devices increases, the tasks must be processed faster and if the system is unable to provide the corresponding services under the limited conditions, the user satisfaction will drop. Figure 7 demonstrates that there are slight variations in the satisfaction from the different methods. The ASTP method has the highest satisfaction, which choose the best computing mode to execute the tasks. Because ASCM is lacking the task execution sequence optimization, the execution sequence still adopts the traditional method and the system satisfaction cannot be increased. With the number of devices increasing, the satisfaction of RSTP and RSCM drop quickly and when the number of devices increases to 100, the satisfaction is reduced to approximately 60%.

## 6. Conclusions

In this paper, we study the adaptive computing optimization problem in an IIoT enable fog computing system for various tasks processing. We propose a Software-Defined IIoT architecture that can realize computing mode adaptive selection in fog computing platform. Then, under the proposed framework, an efficient CMS module is designed in the SDN controller. The task computing delays under different computing modes are calculated in the controller and the controller can select an optimal computing mode for each task. Finally, to ensure the real-time processing of the tasks that choose the fog computing mode, an advanced task execution sequence adjustment mechanism is made according to the task priorities. The proposed method is validated by simulation. Performance evaluations demonstrate that proposed method can greatly improve the real-time performance, reliability and satisfaction in industrial manufacturing. The future research direction is to provide computing services continuously for mobile devices, with complex task decomposition and collaborative computing of fog nodes.

## Figures and Tables

**Table 1:** Parameter values of the simulation.

Parameters	Description	Value
*N*	number of terminal devices	[50, 100]
*M*	number of edge servers	1
*f_n_*	computing capacity of *n*th device	[0.5, 1] G cycles/s
*f_C_*	computing capacity of cloud	8 G cycles/s
*f_E_*	computing capacity of fog server	2 G cycles/s
*D_n_*	data size of the *n*th task	[10, 50] Mb
*P_n,E_*	transmission power	[0.2, 0.6] W
*P_n,max_*	maximum transmission power	0.6 W
*B*	link bandwidth	100 MHz
*μ*_1_	weight of real-time intensity	0.7
*μ*_2_	weight of complex intensity	0.3
*θ_n_*	allocated ratio of bandwidth	[0, 1]

**Table 2:** Applications and parameters.

Applications	*T_i,max_*	*C_i_*	*p_i_*
Process monitoring	10 ms	90 Mcps	0.1191
Environmental monitoring	50 ms	85 Mcps	0.2476
Fault diagnosis	20 ms	50 Mcps	0.1143
Product testing	50 ms	20 Mcps	0.1857
Inventory management	80 ms	70 Mcps	0.3333

## References

[1] Wan J., Yi M., Li D., Zhang C., Wang S., Zhou K. (2016). Mobile Services for Customization Manufacturing Systems: An Example of Industry 4.0. IEEE Access.

[2] Li X., Li D., Wan J., Vasilakos A.V., Lai C.F., Wang S. (2017). A review of industrial wireless networks in the context of Industry 4.0. Wirel. Netw..

[3] Wan J., Tang S., Shu Z., Li D., Wang S., Imran M., Vasilakos A.V. (2016). Software-Defined Industrial Internet of Things in the Context of Industry 4.0. IEEE Sens. J..

[4] Fernández-Caramés T.M., Fraga-Lamas P., Suárez-Albela M., Díaz-Bouza M.A. (2018). A Fog Computing Based Cyber-Physical System for the Automation of Pipe-Related Tasks in the Industry 4.0 Shipyard. Sensors.

[5] Lavassani M., Forsström S., Jennehag U., Zhang T. (2018). Combining Fog Computing with Sensor Mote Machine Learning for Industrial IoT. Sensors.

[6] Delsing J. (2017). Local cloud internet of things automation: Technology and business model features of distributed internet of things automation solutions. IEEE Ind. Electron. Mag..

[7] Beier G., Niehoff S., Xue B. (2018). More Sustainability in Industry through Industrial Internet of Things?. Appl. Sci..

[8] Gungor V.C., Hancke G.P. (2009). Industrial Wireless Sensor Networks: Challenges, Design Principles, and Technical Approaches. IEEE Trans. Ind. Electron..

[9] Wan J., Tang S., Hua Q., Li D., Liu C., Lloret J. (2017). Context-Aware Cloud Robotics for Material Handling in Cognitive Industrial Internet of Things. IEEE Internet Things J..

[10] Linthicum D.S. (2016). The Technical Case for Mixing Cloud Computing and Manufacturing. IEEE Cloud Comput..

[11] Georgakopoulos D., Jayaraman P.P., Fazia M., Villari M., Ranjan R. (2016). Internet of Things and Edge Cloud Computing Roadmap for Manufacturing. IEEE Cloud Comput..

[12] Tao F., Cheng Y., Xu L.D., Zhang L., Li B.H. (2014). CCIoT-CMfg: Cloud Computing and Internet of Things-Based Cloud Manufacturing Service System. IEEE Trans. Ind. Inform..

[13] Li X., Wan J. (2018). Proactive caching for edge computing-enabled industrial mobile wireless networks. Future Gener. Comput. Syst..

[14] Wan J., Chen B., Wang S., Xia M., Li D., Liu C. (2018). Fog Computing for Energy-aware Load Balancing and Scheduling in SmartFactory. IEEE Trans. Ind. Inform..

[15] Yang S. (2017). IoT Stream Processing and Analytics in the Fog. IEEE Commun. Mag..

[16] Ashjaei M., Bengtsson M. Enhancing smart maintenance management using fog computing technology. Proceedings of the 2017 International Conference on Industrial Engineering and Engineering Management.

[17] Yu W. (2018). A Survey on the Edge Computing for the Internet of Things. IEEE Access.

[18] Chiang M., Zhang T. (2016). Fog and IoT: An Overview of Research Opportunities. IEEE Internet Things J..

[19] Mouradian C., Naboulsi D., Yangui S., Glitho R.H., Morrow M.J., Polakos P.A. (2018). A Comprehensive Survey on Fog Computing: State-of-the-Art and Research Challenges. IEEE Commun. Surv. Tutor..

[20] Shi W., Cao J., Zhang Q., Li Y., Xu L. (2016). Edge Computing: Vision and Challenges. IEEE Internet Things J..

[21] Wang J., Li D. (2018). Research and Analysis of Computing Modes in Industrial Internet of Things. Int. J. Auton. Adapt. Commun. Syst..

[22] Li X., Li D., Wan J., Liu C., Imran M. (2018). Adaptive Transmission Optimization in SDN-Based Industrial Internet of Things with Edge Computing. IEEE Internet Things J..

[23] Fu J., Liu Y., Chao H.C., Bhargava B., Zhang Z. (2018). Secure Data Storage and Searching for Industrial IoT by Integrating Fog Computing and Cloud Computing. IEEE Trans. Ind. Inform..

[24] Muhammad G., Alhamid M.F., Alsulaiman M., Gupta B. (2018). Edge Computing with Cloud for Voice Dissequence Assessment and Treatment. IEEE Commun. Mag..

[25] Taleb T., Dutta S., Ksentini A., Iqbal M., Flinck H. (2017). Mobile Edge Computing Potential in Making Cities Smarter. IEEE Commun. Mag..

[26] Liu J., Wan J., Zeng B., Wang Q., Song H., Qiu M. (2017). A Scalable and Quick-Response Software Defined Vehicular Network Assisted by Mobile Edge Computing. IEEE Commun. Mag..

[27] Cao Y., Song H., Kaiwartya O., Zhou B., Zhuang Y., Cao Y., Zhang X. (2018). Mobile Edge Computing for Big-Data-Enabled Electric Vehicle Charging. IEEE Commun. Mag..

[28] Meng X., Wang W., Zhang Z. (2017). Delay-Constrained Hybrid Computation Offloading with Cloud and Fog Computing. IEEE Access.

[29] Liu J., Zhang Q. (2018). Offloading Methods in Mobile Edge Computing for Ultra-Reliable Low Latency Communications. IEEE Access.

[30] Shih Y.Y., Chung W.H., Pang A.C., Chiu T.C., Wei H.Y. (2017). Enabling Low-Latency Applications in Fog-Radio Access Networks. IEEE Netw..

[31] Hu P., Ning H., Qiu T., Zhang Y., Luo X. (2017). Fog Computing Based Face Identification and Resolution Method in Internet of Things. IEEE Trans. Ind. Inform..

[32] Baktir A.C., Ozgovde A., Ersoy C. (2017). How Can Edge Computing Benefit From Software-Defined Networking: A Survey, Use Cases, and Future Directions. IEEE Commun. Surv. Tutor..

[33] Bi Y., Han G., Lin C., Deng Q., Guo L., Li F. (2018). Mobility Support for Fog Computing: An SDN Approach. IEEE Commun. Mag..

[34] Debrito M.S., Hoque S., Steinke R., Willner A., Magedanz T. (2017). Application of the Fog computing paradigm to Smart Factories and cyber-physical systems. Trans. Emerg. Telecommun. Technol..

[35] Mubeen S., Nikolaidis P., Didic A., Pei-Breivold H., Sandström K., Behnam M. (2017). Delay Mitigation in Offloaded Cloud Controllers in Industrial IoT. IEEE Access.

[36] Du J.B., Zhao L.Q., Feng J., Chu X.L. (2018). Computation Offloading and Resource Allocation in Mixed Fog/Cloud Computing Systems with Min-Max Fairness Guarantee. IEEE Trans. Commun..

